# Effects of family relationship and social support on the mental health of Chinese postpartum women

**DOI:** 10.1186/s12884-022-04392-w

**Published:** 2022-01-25

**Authors:** Weijing Qi, Yan Liu, Huicong Lv, Jun Ge, Yucui Meng, Nan Zhao, Fuqing Zhao, Qing Guo, Jie Hu

**Affiliations:** 1 Clinical Humanistic Care and Nursing Research Center, Nursing School, Hebei Medical University, 361 East Zhongshan Road, Shijiazhuang, 050017 Hebei Province China; 2 Obstetrics and Gynecology, Peking University People’s Hospital, Beijing, China; 3 Shijiazhuang Obstetrics and Gynecology Hospital, 206 East Zhongshan Road, Shijiazhuang, 050017 Hebei Province China

**Keywords:** Social support, Postpartum depression, Sleep quality, Family relationship

## Abstract

**Background:**

Numerous studies suggest that interpersonal relationships and social support influence the development of postpartum depression and sleep quality for women. However, the effect of support from the husband or the mother-in-law has not been thoroughly validated. The current study examined the relative contribution of marital satisfaction, perceived caring of the mother-in-law, and social support on postpartum depression and sleep quality simultaneously in a path model.

**Methods:**

A cross-sectional study was conducted from March to June 2017 in Hebei, China, using a self-report questionnaire. A total of 817 women participated at 6 weeks postpartum. Sociodemographics and information about marital satisfaction, perceived caring of the mother-in-law, social support, postpartum depression, and sleep were collected. Path analysis was used to analyze the cross-sectional data.

**Results:**

The final model had a highly satisfactory fit. Marital satisfaction and perceived caring of mother-in-law had both direct and indirect effects on postpartum depression through social support, but these two variables had only an indirect effect on sleep quality through social support and postpartum depression. Sleep quality is a consequence of postpartum depression at 6 weeks after delivery. The prevalence of minor and major postpartum depressive symptoms at 6 weeks postpartum was 41.49 and 23.13%, respectively. A total of 371 (45.41%) women experienced sleep disturbance at 6 weeks postpartum.

**Conclusions:**

These findings suggest that interpersonal relationships with family members play important roles in postpartum depression and sleep quality through social support in Chinese women. Improving the relationship between new mothers and their husbands or mothers-in-law and then enhancing social support might reduce postpartum depression and sleep disturbance.

## Background

Delivery involves major psychological and social changes in women. These changes may lead to postpartum blues and even postpartum depression (PPD). Distinguished from postpartum blues and postpartum psychosis, PPD is defined as an episode of major depressive disorder or sometimes minor depression that occurs in the postpartum period [1]. The effects of PPD are most often seen in women with chronic ongoing and untreated depression. The significant and enduring negative effect of maternal PPD on children has been well documented [2]. For example, higher depressive symptoms of the mother are associated with lower infant weight and increased infant problems with physical health and sleep [3]. Additionally, child cognitive development, such as language and IQ, and some aspects of behavioral performance may be influenced by the mother’s PPD [4]. However, considerable evidence exists that with adequate treatment; the effects of depression are largely mitigated or avoided. A recent review of the magnitude of PPD illustrated that the prevalence of PPD in developed countries ranges from 1.9 to 82.1% and in developing countries from 5.2 to 74.0% [5]. In China, the prevalence of PPD is 31.6% at 1 week and 6.7% at 1 month after delivery [6]. Given the lasting adverse effects and high prevalence of PPD, great public and professional concern has developed and prompted a call for research to identify risk factors to inform future intervention targets.

Increasing evidence indicates that psychosocial factors have moderate-to-strong effect sizes in PPD. Among these factors, except for prenatal factors such as antenatal depression, anxiety, and previous psychiatric illness [7–10], lack of social support and bad interpersonal relationships, especially marital relationships, are the strongest risk factors for PPD. Delivery is a major life event for new mothers; it means leaving work or school, coping with the needs and problems of baby, suffering from physical pains, and lacking time for social activities. Therefore, social support, especially from family members, is a powerful aid for these women. Many researchers have investigated the relationship between social support and PPD. Almost all of them found that lack of social support was negatively correlated with PPD [11–15].

In China, after delivery, women take maternity leave for approximately 5 months. During this period, the husband is the primary support provider. Support from husbands, especially emotional support, is needed for Chinese women after delivery [16]. Similarly, a prospective study conducted in China also found that postpartum depressive symptomatology was determined by marital dissatisfaction [17].

In addition to the husband, the mother-in-law is another main support provider for postpartum women [18, 19]. Low social support from mothers-in-law has been found to be associated with PPD all over the world [11, 20, 21]. Our previous qualitative study also found that conflicts regarding details of baby care, differences in lifestyle between a woman and her mother-in-law, and excessive interference from the mother-in-law caused negative moods in postpartum women [22]. Thus, we suggest that postpartum mothers’ perceived support from their mothers-in-law and their marital satisfaction may predict their social support and that social support may predict PPD.

In addition to depression, sleep quality is another important concern for postpartum women. Many studies have reported a significant positive relationship between sleep quality and depression after delivery [23–25]. For example, Okun reviewed the studies about relationship of disturbed sleep during the perinatal period and PPD, and summarized that sleep disturbance during that period is significantly associated with an increased risk of depression [25]. Nevertheless, the existence of a causal relationship between sleep quality and depression is controversial [26]. Some studies have found that sleep quality predicts depression, while others have found that depression is a risk factor for sleep quality [27]. In this study, we hypothesized that sleep quality is a symptom and consequence of depression and vice versa.

**Fig. 1:**
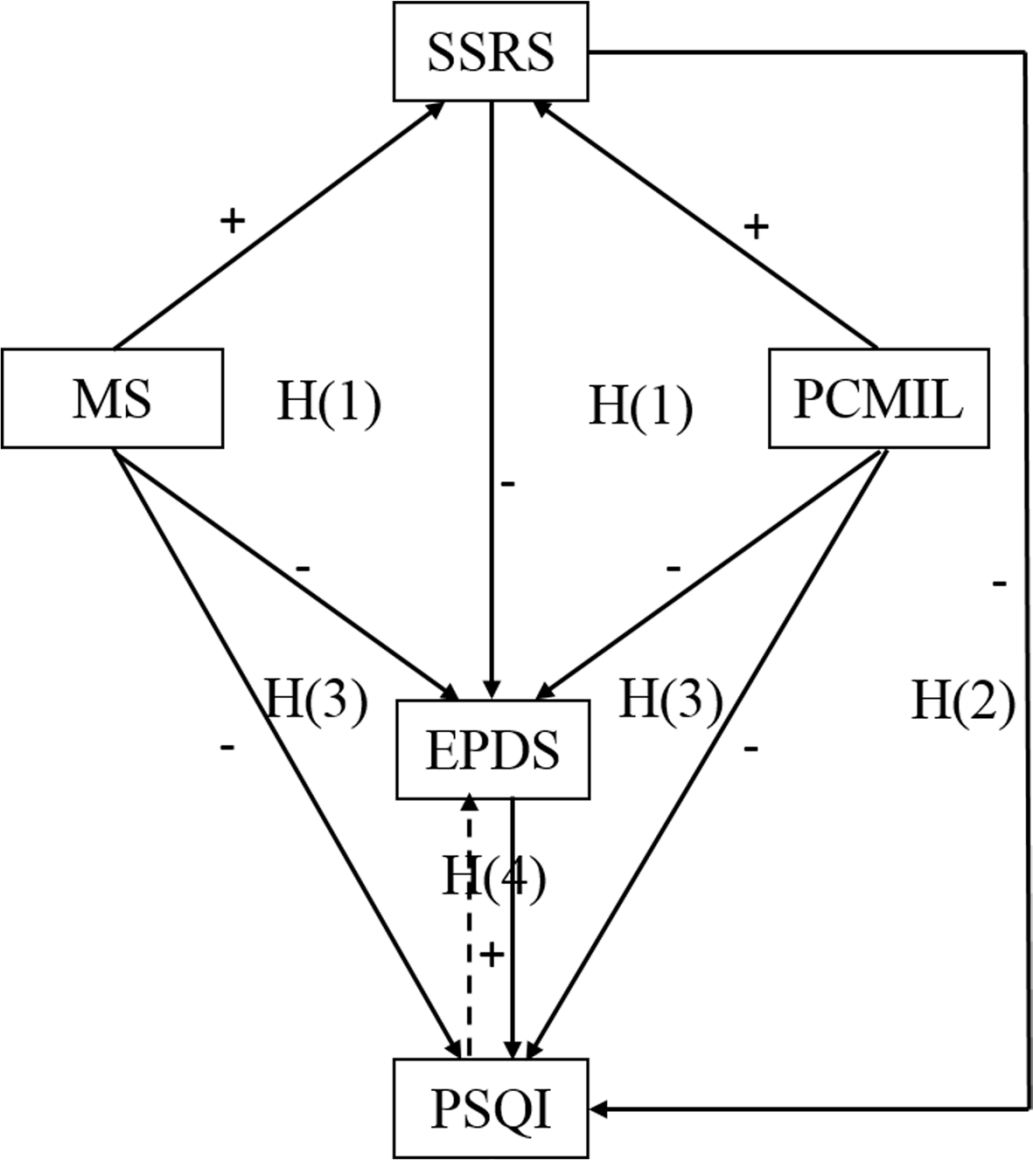
Model 1—Hypothesized model. SSRS = Social Support Rating Scale, MS = Marital Satisfaction, PCMIL = Perceived caring of Mother-in-law, EPDS = Edinburgh Postnatal Depression Scale, PSQI = Pittsburgh Sleep Quality Index, H(1)(2)(3)(4) = hypotheses(1)(2)(3)(4)

Although a considerable amount of research has demonstrated interrelations between two of these variables (social supports, interpersonal relationships with family members, sleep disturbance, and PPD), few studies have investigated the combined effects of social support from husband (peer relationship) and mother-in-law (intergenerational relationship) on PPD and sleep quality, and the direct and indirect effects of these factors. The purpose of the current study was to examine the combined effects of marital satisfaction, perceived caring of the mother-in-law, and social support on PPD and sleep quality in a path model. As depicted in Fig. 1, based on previous studies, we hypothesized the following: (1) marital satisfaction or perceived caring of the mother-in-law will have a direct effect on PPD and an indirect effect on PPD through social support; (2) social support will have a direct relationship with PPD or sleep quality and an indirect relationship with sleep quality through PPD; (3) marital satisfaction or perceived caring of the mother-in-law will have a direct effect on sleep quality and an indirect effect on sleep quality through social support and PPD; and (4) PPD and sleep quality will have a bidirectional relationship at 6 weeks postpartum.

## Methods

### Participants and procedures

This cross-sectional study was conducted in Shijiazhuang, Hebei Province, China, from March to June 2017. A sample of 887 postpartum women was recruited from Shijiazhuang Obstetrics and Gynecology Hospital. Eligible participants were those who 1) delivered in the past 6 weeks, 2) had a live birth, and 3) had no history of mental illness or brain disease.

All participants were registered before delivery in a fetal monitor room where they were informed about the study purpose. Our survey workers followed up all registered participants at 6 weeks postpartum through WeChat, and distributed questionnaires to those who met the above inclusion criteria through an online crowdsourcing platform in mainland China, which provides functions equivalent to Amazon Mechanical Turk. Informed consent was obtained from participants when they registered before delivery. Each participant received a small gift for her participation. Ethical approval was obtained from the hospital’s Ethics Committee and the Ethics Review Boards at Hebei Medical University (Ethic code: 2016021).

### Measures

#### Sociodemographic characteristics

The sociodemographic characteristics of the participants were collected at baseline including residence, age, educational level, parity, accidental pregnancy.

#### Postpartum depression

The Edinburgh Postnatal Depression Scale (EPDS) is widely used to measure depression symptoms in women after delivery [28]. This self-report instrument consists of 10 items, with each item scored from 0 to 3 (range 0–30). The Chinese version of the EPDS has been validated in samples of Chinese women during the postpartum period [29], with well-documented reliability (internal consistency of 0.87) and validity (concurrent validity with the Beck Depression Inventory of 0.79) [30]. A sensitivity of 81.25% and specificity of 80.67% (using a cut-off score of 10.5) of the scale administered during the postnatal period have been demonstrated [31]. In this study, the EPDS showed high internal consistency, with a Cronbach’s alpha of 0.865. The prevalence of minor and major depression after delivery was identified by using the recommended cut-off scores of 10 and 13, respectively [28, 30].

#### Sleep quality

The Pittsburgh Sleep Quality Index (PSQI) is a 19-item self-report questionnaire to assess sleep disturbance over the past 1 month [32, 33]. The Chinese version of the PSQI has been proven to have excellent internal consistency and validity [34]. Seven component scores are computed from the items: subjective sleep quality, sleep latency, sleep duration, habitual sleep efficiency, sleep disturbances, sleep medication, and daytime dysfunction. The scores of these components range from 0 (no difficulty) to 3 (severe difficulty). The sum of the seven component scores yields a global sleep quality score (range 0–21), with a higher score denoting poorer sleep quality. According to the recommendations of previous studies [35, 36], a cut-off total score of 8 was used as the threshold of sleep disturbance symptoms. In the present study, the PSQI had a Cronbach’s alpha of 0.700, indicating an acceptable level of reliability.

#### Marital satisfaction

The degree of marital satisfaction reported by the study participants was estimated by a marriage questionnaire that consisted of 28 items adapted from the Burgess and Wallin marriage success index [37]. The instrument was proved with good validity and reliability in China [38]. For each item, participants were asked to rate their marital satisfaction using a scale of 1 (strongly disagree) to 4 (strongly agree). A total score was calculated by computing the sum of the 28 items, with a higher total score indicating higher marital satisfaction. In the current sample, the internal consistency was excellent for the total scale (Cronbach’s alpha = 0.901).

#### Perceived caring of mother-in-law

Perceived caring of mother-in-law was evaluated by asking the respondents to rate “the degree of caring to you from your mother-in-law” on a scale ranging from 1 to 10, with higher scores indicating higher perceived caring of the mother-in-law.

#### Social support

The Social Support Rating Scale (SSRS) was used to assess participants’ social support; the SSRS was developed by Xiao for the Chinese environmental and cultural context [39]. It consists of 10 items evaluating three domains: subjective support (4 items), objective support (3 items), and support availability (3 items). The total score is the sum of the scores for each item, with a higher score indicating better social support. This scale has been widely used in different Chinese populations and has been shown to be valid and reliable [40]. In the present sample, the internal consistency was excellent for the total scale (Cronbach’s alpha = 0.706).

#### Data analysis

Data were analyzed using SPSS 25.0 (SPSS Inc., Chicago, IL, USA), and the path analysis was run using Mplus 7.0. Descriptive statistics were used to describe the sample characteristics. Frequencies and percentages were calculated to describe the baseline characteristics. Chi-square test was used to detect the difference of number of EPDS> = 10 or PSQI> = 8 at subsample level. Means and standard deviations (SDs) were calculated to describe the study variables. Bivariate Pearson correlation coefficients were used to examine the relationship among marital satisfaction, caring of mother-in-law, social support dimensions, sleep quality dimensions, and PPD. Then, the path analysis was conducted using maximum likelihood estimation and the bootstrap method (1000 replicates) to test the hypothesized relationships among the above variables. The hypothesized model is shown in Fig. 1. Path analysis facilitates the investigation of direct and indirect effects between variables. Several indices, such as chi-square (χ2), χ2/df, root mean square error of approximation (RMSEA), comparative fit index (CFI), the Tucker-Lewis Index (TLI), and standardized root mean square residual (SRMR), were used to determine whether the hypothesized model fit the observed data. The model fit was considered adequate when the χ2 was not significant, χ2/df was less than 2.0, the CFL and TLI were over 0.90, and the MSEA and SRMR were less than 0.05 [41, 42]. The *P* value was significant at 0.05.

## Results

### Sociodemographic characteristics

Among the 887 women, 817 completed all the questionnaires, with a 92.11% response rate. Table 1 reveals the sociodemographic characteristics of the 817 women who completed all the questionnaires. Most of the participants lived in urban (*n* = 747, 91.43%), while only 8.57% lived in rural. Nearly three-fifths (*n* = 479, 58.63%) were 30 years old and younger, and the rest were 31 and older. Most had an undergraduate level of education (*n* = 566, 69.28%). Slightly more than half (*n* = 457, 55.94%) were primiparas. Approximately one-third of them reported their pregnancy to be unplanned. There was a significant difference of minor PPD among participants of diverse education levels, indicating that post-graduate women had a higher rate (51.76%) of depression at 6 weeks after delivery.

**Table 1:** Sociodemographic characteristics of participants (*n* = 817)

	n	%	# of EPDS> = 10 at subsample level	χ2	# of PSQI> = 8 at subsample level	χ2
Residence						
Rural	70	8.57	27(38.57%)	0.269	33(47.14%)	0.093
Urban	747	91.43	312(41.77%)		338(45.25%)	
Age (year)						
< =30	479	58.63	211(44.05%)	3.118	220(45.93%)	0.126
> =31	338	41.37	128(37.87%)		151(44.67%)	
Educational level						
Junior/Senior school	166	20.32	76(45.78%)	6.781^*^	76(45.78%)	0.137
Undergraduate	566	69.28	219(38.69%)		258(45.58%)	
Post-graduate	85	10.4	44(51.76%)		37(43.53%)	
Parity						
Primiparous	457	55.94	192(42.01%)	0.115	205(44.88%)	0.128
Multiparous	360	44.06	147(40.83%)		166(46.11%)	
Accidental pregnancy						
Yes	214	26.19	98(45.79%)	2.210	90(42.06%)	1.316
No	603	73.81	241(39.97%)		281(46.60%)	

^*^p < 0.05

### Prevalence of postpartum depression and sleep disturbance

The prevalence of minor and major depressive symptoms at 6 weeks postpartum was 41.49% (*n* = 339, EPDS > = 10, 95% CI = 38.12, 44.86%) and 23.13% (*n* = 189, EPDS > = 13, 95% CI = 20.23, 26.03%), respectively. A total of 371 (45.41%, PSQI > = 8, 95% CI = 42, 48.82%) women experienced sleep disturbance at 6 weeks postpartum.

### Correlational analysis

The means and standard deviations of all study variables and the bivariate correlations among all variables included in the hypothesized model are reported in Table 2. Sleep quality was significantly positively correlated with PPD. There were significant positive correlations between marital satisfaction or perceived caring of mother-in-law and social support, and significant negative correlations between marital satisfaction or perceived caring of mother-in-law and PPD or sleep quality. Social support was significantly negatively correlated with PPD or sleep quality. All correlations were significant at the *p* < 0.01 level.

**Table 2:** Means, standard deviations, and the correlation of study variables

	M	SD	EPDS	PSQI	MS	PCMIL
EPDS	8.58	5.49				
PSQI	7.35	3.59	.516^**^			
MS	84.56	9.56	−.442^**^	−.301^**^		
PCMIL	6.85	2.88	−.309^**^	−.227^**^	.296^**^	
SSRS	38.42	5.58	−.455^**^	−.357^**^	.412^**^	.325^**^

Notes: *EPDS* Edinburgh Postnatal Depression Scale, *M* mean, *MS* Marital Satisfaction, *PCMIL* Perceived caring of Mother-in-law, *PSQI* Pittsburgh Sleep Quality Index, *SD* standard deviations, *SSRS* Social Support Rating Scale

*n* = 817

^*^p < 0.05

^**^p < 0.01

### Model testing

Path modeling failed to identify the full initial model because there were two hypothesized paths among the uncorrelated variables. Subsequent models were constructed and trimmed by comparing the standardized beta coefficients and fit indices. Contrary to the hypotheses, marital satisfaction and caring of mother-in-law did not have direct relationships with sleep quality.

**Fig. 2:**
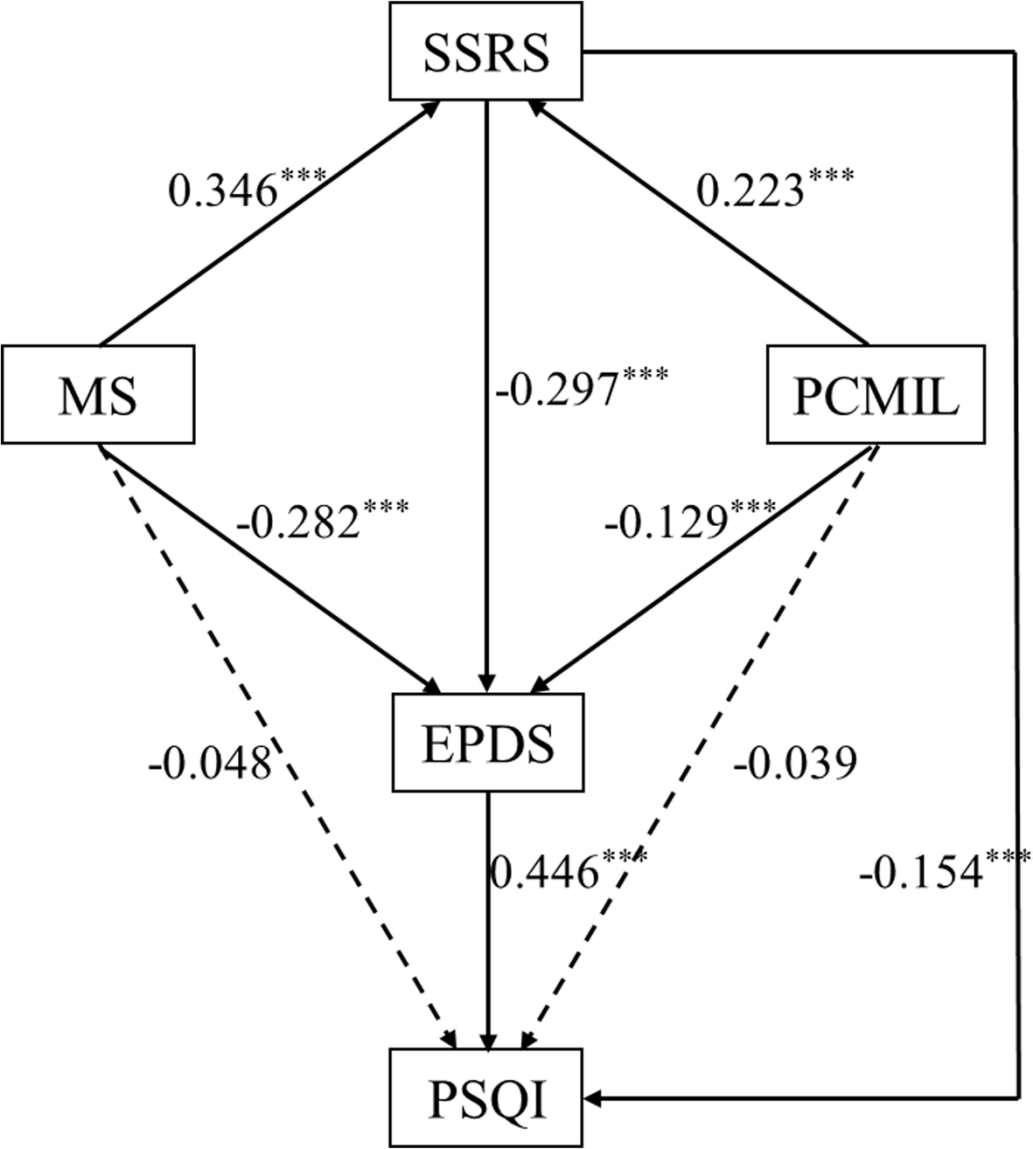
Model 2—Final model for the whole sample (*N* = 817), with standardized beta weights and significance levels. Fit statistics: χ2 = 3.986, *P* = 0.136; RMSEA = 0.035; CFI = 0.997;TLI = 0.988; SRMR = 0.013. SSRS = Social Support Rating Scale, MS = Marital Satisfaction, PCMIL = Perceived caring of Mother-in-law, EPDS = Edinburgh Postnatal Depression Scale, PSQI = Pittsburgh Sleep Quality Index

The final simplified model (Fig. 2) demonstrated an excellent fit: χ^2^ = 3.986, *P* = 0.136, χ^2^ /*df* = 1.993, RMSEA = 0.035, CFI = 0.997, TLI = 0.988, SRMR = 0.013. In order to validate the directional relationship of PPD and sleep quality, we exchanged the position of PSQI and EPDS in the final model. A poor fit to the data was indicated by the following statistics: χ^2^ /*df* = 35.42, *P* = 0.000, RMSEA = 0.205, CFI = 0.909, TLI = 0.591, SRMR = 0.050, indicating that PPD was a predictor of sleep quality.

The standardized regression coefficients and standard errors of the final model are summarized in Table 3. The model is interpreted as follows: First, marital satisfaction (β = − 0.282, bootstrap 95% CI = − 0.350, − 0.216, *p* = 0.000), perceived caring of mother-in-law (β = − 0.129, bootstrap 95% CI = − 0.197, − 0.061, *p* = 0.000) and social support (β = − 0.297, bootstrap 95% CI = − 0.357, − 0.221, *p* = 0.000) were directly predictive of PPD. Second, social support (β = − 0.154, bootstrap 95% CI = − 0.219, − 0.080, *p* = 0.000) and PPD (β = 0.446, bootstrap 95% CI = 0.389, 0.512, *p* = 0.000) were directly predictive of sleep quality. Third, marital satisfaction (β = 0.346, bootstrap 95% CI = 0.284, 0.409, *p* = 0.000) and perceived caring of mother-in-law (β = 0.223, bootstrap 95% CI = 0.151, 0.283, *p* = 0.000) were associated with higher social support (Table 3).

**Table 3:** Unstandardized and Standardized regression coefficients and standard errors for all pathways of the final model (*n* = 817)

Variable 1	Variable 2	B	β	SE	p	95%CI
MS	EPDS	− 0.162	− 0.282	0.035	0.000	−0.350, − 0.216
PCMIL	EPDS	− 0.245	− 0.129	0.034	0.000	− 0.197, − 0.061
SSRS	EPDS	− 0.292	− 0.297	0.034	0.000	− 0.357, − 0.221
SSRS	PSQI	− 0.099	− 0.154	0.035	0.000	− 0.219, − 0.080
EPDS	PSQI	0.292	0.446	0.032	0.000	0.389, 0.512
MS	SSRS	0.202	0.346	0.031	0.000	0.284, 0.409
PCMIL	SSRS	0.431	0.223	0.034	0.000	0.151, 0.283

Notes: *EPDS* Edinburgh Postnatal Depression Scale, *MS* Marital Satisfaction, *PCMIL* Perceived caring of Mother-in-law, *PSQI* Pittsburgh Sleep Quality Index, *SSRS* Social Support Rating Scale

### Direct, indirect, and total effects on PPD and sleep quality

The standardized direct and indirect effects are reported in Table 4. Marital satisfaction demonstrated both a direct (β = − 0.282, bootstrap 95% CI = − 0.350, − 0.216, *p* = 0.000) and an indirect effect (β = − 0.103, bootstrap 95% CI = − 0.132, − 0.073, *p* = 0.000) on PPD through social support, and it had the greatest accumulated total effect on PPD (β = − 0.385, bootstrap 95% CI = − 0.449, − 0.321, *p* = 0.000). Perceived caring of mother-in-law also illustrated both a direct (β = − 0.129, bootstrap 95% CI = − 0.197, − 0.061, *p* = 0.000) and an indirect effect (β = − 0.066, bootstrap 95% CI = − 0.094, − 0.042, *p* = 0.000) on PPD through social support, and it had the weakest accumulated total effect on PPD (β = − 0.195, bootstrap 95% CI = − 0.258, − 0.124, *p* = 0.000). Social support had a direct effect on PPD (β = − 0.297, bootstrap 95% CI = − 0.357, − 0.221, *p* = 0.000).

**Table 4:** Standardized direct, indirect, and total effect of all study pathways (*n* = 817)

Pathway	Direct effect β(95% CI)	Indirect effect β(95% CI)	Total effect β(95% CI)
**MS→EPDS**	−0.282(−0.350, −0.216)	− 0.103(− 0.132, − 0.073)	−0.385(− 0.449, − 0.321)
**PCMIL→EPDS**	−0.129(− 0.197, − 0.061)	−0.066(− 0.094, − 0.042)	−0.195(− 0.258, − 0.124)
**SSRS→EPDS**	−0.297(− 0.357, − 0.221)	–	−0.297(− 0.357, − 0.221)
**EPDS→PSQI**	0.446(0.389, 0.512)	–	0.446(0.389, 0.512)
**SSRS→PSQI**	−0.154(− 0.219, − 0.080)	−0.132(− 0.171, − 0.097)	−0.287(− 0.353, − 0.221)
**MS→PSQI**	–	− 0.179(− 0.217, − 0.143)	−0.179(− 0.217, − 0.143)
**PCMIL→PSQI**	–	− 0.092(− 0.125, − 0.060)	−0.092(− 0.125, − 0.060)

Notes: *EPDS* Edinburgh Postnatal Depression Scale, *MS* Marital Satisfaction, *PCMIL* Perceived caring of Mother-in-law, *PSQI* Pittsburgh Sleep Quality Index, *SSRS* Social Support Rating Scale

For sleep quality, PPD demonstrated a direct and strongest total effect (β = 0.446, bootstrap 95% CI = 0.389, 0.512, *p* = 0.000) on sleep quality. Social support showed both a direct (β = − 0.154, bootstrap 95% CI = − 0.219, − 0.080, *p* = 0.000) and an indirect effect (β = − 0.132, bootstrap 95% CI = − 0.171, − 0.097, *p* = 0.000) on sleep quality through PPD, and it had the second strongest total effect (β = − 0.287, bootstrap 95% CI = − 0.353, − 0.221, *p* = 0.000). Both marital satisfaction (β = − 0.179, bootstrap 95% CI = − 0.217, − 0.143, *p* = 0.000) and perceived caring of mother-in-law (β = − 0.092, bootstrap 95% CI = − 0.125, − 0.060, *p* = 0.000) had only an indirect effect on sleep quality through social support and PPD.

## Discussion

We investigated in the present study the associations between marital satisfaction, perceived caring of mother-in-law, social support, sleep quality, and PPD by using path analysis to identify the best combination of social support factors that contribute to PPD and sleep quality. We found that marital satisfaction or perceived caring of mother-in-law had a direct effect on PPD and an indirect effect on PPD through social support (consistent with hypothesis 1); social support had a direct relationship with PPD or sleep quality and an indirect relationship with sleep quality through PPD (consistent with hypothesis 2); marital satisfaction or perceived caring of mother-in-law only had an indirect effect on sleep quality through social support and PPD (partly consistent with hypothesis 3); and PPD was a predictor of sleep quality at 6 weeks postpartum (partly consistent with hypothesis 4). Overall, the findings in this study indicated that the model had an excellent fit. These study results revealed the important roles of social support from the women’s family members on PPD and sleep quality during postpartum.

Consistent with our hypothesis 1 in the present study, marital satisfaction was not only directly associated with PPD but also had an indirect effect on PPD through social support. And, we found that the total effect of marital satisfaction on PPD was larger than that of the other variables. This finding verified the results of previous studies conducted in different culture contexts, which highlighted that low marital satisfaction was an important predictive variable of PPD [43–47]. Pregnant women with a higher marital satisfaction had a significantly lower risk for the development of PPD [48]. Postpartum marital satisfaction can contribute to perceived social support, which subsequently decreases depression [49]. However, new parents perceived decreased caring and relationship satisfaction with each other compared to that before their children were born [50]. In Chinese culture, weakening emotional and instrumental involvement in taking care of babies for new fathers who have to earn more money to support their wives during postpartum may result in less marital satisfaction and more marital conflict [51]. Thus, the couple’s relationship is a very important concern for postpartum women, for increasing marital satisfaction and perceived social support of postpartum women could prevent development or deterioration of PPD. Couple-focused interventions [52] or a program of promoting the father’s involvement in caregiving [53] are needed in the early postpartum period or even earlier.

In China, in addition to husbands, mothers-in-law are primary caregivers of the postpartum women. Thus, it is not surprising that perceived caring of the mother-in-law was also found to be a significant predictor of PPD. The results are consistent with prior study results indicating that a woman’s interaction with her mother-in-law during the “confinement” period is an important aspect that could contribute to or fail to protect against PPD [19, 54, 55]. Our results further illustrated effects of perceived caring of the mother-in-law on PPD included two paths as well as marital satisfaction, direct effect and indirect effect through social support. Therefore, improving relationships between new mothers with their husbands or mothers-in-law, not only plays an important role in directly reducing PPD, but also can indirectly prevent PPD via increasing social support. Although numerous studies have confirmed the efficacy of interpersonal psychotherapy (IPT) for PPD in Western countries [56–59], few such studies have been conducted in China. Based on our results that family interpersonal relationships are the main resources of social support for postpartum women, how to make IPT adapt to Chinese family culture is a future research direction.

In this study, a self-formulated question was used to measure the relationship between new mothers and their mothers-in-law. Firstly, according to our previous qualitative study [22], this variable negatively affects the level of postpartum depression. Other research also suggested that living with parents-in-law may be a risk factor for PPD among Chinese puerperal women [60]. Secondly, in light of the social support theory, the main effect of perceived social support on mental health primarily reflects ordinary social interaction with the providers [61, 62]. If postpartum mothers do not perceive social support subjectively, support is invalid. Therefore, it is necessary to measure the postpartum mothers’ subjective feeling of the relationship between them and their mothers-in-law. However, there is currently no suitable questionnaires or scales to measure this relationship in Chinese culture. Consequently, a self-developed question “Please rate the degree of caring to you from your mother-in-law” was used in this study. This question was designed based on the widely used method to measure pain which is also a subjective feeling [63]. Moreover, the relationship between mother-in-law and daughter-in-law is a warmly-discussed and deep-rooted topic in Chinese culture, and it becomes more sensitive and fragile in perinatal period [19], which means higher tendency for new mothers to express their feelings by this subjective question. In conclusion, this self-formulated question effectively measured the subjective feeling of relationship between participants and their mothers-in-law.

Our findings provide a clearer understanding of the relationship between PPD and sleep quality. Previous considerable research on this relationship has revealed that sleep disturbance predicts PPD [64–66], but there is some controversy about the interactive relationship between them [24, 67]. Our results confirmed that PPD causes sleep disturbance, consistent with some prior study results [23, 68]. One possible explanation for this finding might be that our data were collected at 6 weeks after delivery, when the new mother had adapted to taking care of her new baby. Sleep-related problems at this point were mainly subjective sleep quality and daytime dysfunction, which are affected by subjective mood state and family support, respectively. Thus, sleep disturbance emerged as a consequence of depression, not a cause.

This was a cross-sectional investigation with large sample. Our results revealed the key role of mother-in-law in the mental health of Chinese postpartum women, as well as husband. The present study was the first to illuminate the mutual relationship in a path model among interpersonal relationships with family members, social support, PPD, and sleep quality in postpartum women in China. Based on our clarification of the mechanisms of the psychological paths to PPD and sleep quality, new targeted methods for intervention, such as interpersonal psychotherapy tailored to the Chinese cultural context and enhanced social support, especially emotional support from family members, will be needed. It is necessary for clinical therapy programs for depression among postpartum women to explore general family education plans and specific family therapies in China.

### Limitations

The present study has several limitations: First, perceived caring of mother-in-law was not measured using a mature scale; a more scientific measurement could show more reliability. Second, most of our sample came from urban, so the results could not be generalized to rural populations in China. Further research could include more postpartum women from rural. Finally, postpartum stress has been reported to be a major risk factor for PPD [69], and it may be a trigger or mediator in the relationship between social support and depression or sleep disturbance. Thus, postpartum stress should be considered as a variable in this path model in future research.

## Conclusion

This study finding reinforces the importance of interpersonal relationships and social support in families for postpartum women. The role of mothers-in-law is the key variable in the final model, the same as the role of husbands. The effects of interpersonal relationships with mothers-in-law and husbands on depression or sleep quality were mediated by social support. The present study provided one picture of the complex bidirectional relationship between PPD and sleep quality. Improving the relationship between new mothers and their husbands and mothers-in-law and enhancing social support might reduce PPD and sleep disturbance.

## Data Availability

The datasets used and/or analyzed during the current study available from the corresponding author on reasonable request.

## Abbreviations

PPD: Postpartum depression
EPDS: Edinburgh Postnatal Depression Scale
PSQI: Pittsburgh Sleep Quality Index
SSRS: Social Support Rating Scale
RMSEA: Root mean square error of approximation
CFI: Comparative fit index
TLI: Tucker-Lewis Index
SRMR: Standardized root mean square residual
